# PTPRK Expression Is Downregulated in Drug Resistant Ovarian Cancer Cell Lines, and Especially in ALDH1A1 Positive CSCs-Like Populations

**DOI:** 10.3390/ijms20082053

**Published:** 2019-04-25

**Authors:** Monika Świerczewska, Karolina Sterzyńska, Karolina Wojtowicz, Dominika Kaźmierczak, Dariusz Iżycki, Michał Nowicki, Maciej Zabel, Radosław Januchowski

**Affiliations:** 1 Department of Histology and Embryology, Poznan University of Medical Sciences, Święcickiego 6 St., 61-781 Poznań, Poland; k.olejniczak@ump.edu.pl (K.S.); kwojtowicz@ump.edu.pl (K.W.); dominika.ka.poznan@gmail.com (D.K.); mnowicki@ump.edu.pl (M.N.); mazab@ump.edu.pl (M.Z.); rjanuchowski@ump.edu.pl (R.J.); 2 Department of Cancer Immunology, Poznan University of Medical Sciences, Garbary 15 St., 61-866 Poznań, Poland; dmizy@ump.edu.pl; 3 Department of Anatomy and Histology, University of Zielona Góra, Licealna 9 St., 65-417 Zielona Góra, Poland

**Keywords:** protein tyrosine phosphatase receptor type K (PTPRK), cancer stem cells (CSCs), drug resistance, ovarian cancer

## Abstract

**Background:** Ovarian cancer is the 7th most common cancer and 8th most mortal cancer among woman. The standard treatment includes cytoreduction surgery followed by chemotherapy. Unfortunately, in most cases, after treatment, cancer develops drug resistance. Decreased expression and/or activity of protein phosphatases leads to increased signal transduction and development of drug resistance in cancer cells. **Methods:** Using sensitive (W1, A2780) and resistant ovarian cancer cell lines, the expression of Protein Tyrosine Phosphatase Receptor Type K (PTPRK) was performed at the mRNA (real-time PCR analysis) and protein level (Western blot, immunofluorescence analysis). The protein expression in ovarian cancer tissues was determined by immunohistochemistry. **Results:** The results showed a decreased level of PTPRK expression in ovarian cancer cell lines resistant to cisplatin (CIS), paclitaxel (PAC), doxorubicin (DOX), topotecan (TOP), vincristine (VIN) and methotrexate (MTX). Additionally, the lower PTPRK expression was observed in Aldehyde Dehydrogenase 1 Family Member A1 (ALDH1A1) positive cancer stem cells (CSCs) population, suggesting the role of PTPRK downregulation in primary as well as acquired resistance to cytotoxic drugs. **Conclusions:** These results provide important insights into the role of PTPRK in mechanism leading to drug resistance in ovarian cancer and has raised important questions about the role of imbalance in processes of phosphorylation and dephosphorylation.

## 1. Introduction

Over the past century, there has been a dramatic increase in cancer diseases. Globally, ovarian cancer is the 7th most common cancer and 8th most mortal cancer among woman. The five-year survival rate is below 45% [1]. The epidemiology is still unclear. Some factors like mutation in *BCRA* genes and menopausal hormone therapy increases risk of ovarian cancer [2], while others, like pregnancy and breastfeeding, lower risk of ovarian cancer [1].

Independently of histological type of the ovarian cancer [3] platinum-based chemotherapy in combination with paclitaxel (PAC) or platinum-based therapy alone is the standard of care for first-line [4]. Although therapy is often effective at the beginning of treatment—most of the treated patients show relapses. In the patients not sensitive to platinum and resistant to platinum, additional agents are often used in the second line of chemotherapy, such as topotecan (TOP), liposomal doxorubicin (DOX), or gemcitabine [5]. However, in most cases after treatment, cancers develop drug resistance.

At the cellular level, cancer cells can develop different mechanisms of drug resistance during which the drug cellular localization is changed, drugs are inactivated faster, DNA is repaired quicker or accumulation of the drug in the cancer cell is decreased.

The reversible phosphorylation is one of the major systems which regulates cell functions. Kinases and phosphatases activate/deactivate many receptors and other signaling proteins by phosphorylation and dephosphorylation events, resulting in up/down regulation signals transductions and changes in cellular metabolism, genes expression and rate of cell proliferation [6].

The balance between these two processes is important for regulating metabolism, proliferation, apoptosis, inflammation and other important physiological processes in cells [6]. Alterations in phosphatase expression, localizations and phosphatase mutations may lead to numerous diseases such as: cancer, metabolic and autoimmune disorders, infectious diseases and neurodegeneration and can alter response to therapy [7,8]. Cancer cells usually have a higher level of protein phosphorylation [9] and increase in protein phosphorylation is associated with development of drug resistance [10].

Protein phosphatases can be divided into three families: protein–tyrosine phosphatase (PTP) family, metallo-dependent protein phosphatase (PPM) family and phosphoprotein phosphatase (PPP) family [6]. Among PTP, receptor-like forms and non-receptor forms are distinguished. The non-receptor subfamily is composed of PTP1B, SHP2, and PTPD1 [11]. Among receptor forms. we can distinguish: DEP1, LAR, PTPµ, and PTPRK [11]. PTPs can negatively or positively regulate RTKs (receptor-tyrosine kinases) [11] and act as tumor suppressors or be involved in tumor progression.

PTPRK (PTPκ) belongs to the R2B subfamily [12]. They have three regions: extracellular, transmembrane and intracellular. The extracellular domain is a Cell Adhesion Molecule-like domain (CAM-like domain) allowing cell–cell adhesion [12]. The intracellular part of PTPRK contains two PTP domains: D1 and D2, where D1 is catalytically active [13]. The physiological role of the PTPRK is fulfilled by highly specific intercellular homophilic interactions forming and, in this way, can directly induce cell–cell contact and mediate contact inhibition of cell growth [14].

Some changes in PTPRK expression can have an influence on cancer development. It has been reported that decreased expression of PTPRK correlates with poor prognosis in breast cancer [15]. In nasal-type NK/T-cell lymphoma (NKTCL), loss of PTPRK expression leads to STAT3 activation and NKTCL pathogenesis and decreased overall survival [16]. Mutation in *PTPRK* gene leads to increased chemotherapy resistance in glioma [14].

There are different models which explain tumor development and drug resistance. One of them describes the concept of cancer stem cells (CSC). These cells have several features like normal stem cells: immortality, unlimited proliferation, resistance to the apoptosis, and self-renewal [17]. Additionally, the expression of molecular pumps such as P-glycoprotein (P-gp) and Breast Cancer Resistance Protein (BCRP) and detoxification enzymes as aldehyde dehydrogenases (ALDHs) explain their resistance to the chemotherapy and radiotherapy [17,18,19]. The most universal marker of CSCs in solid tumor is an expression of aldehyde dehydrogenase 1A1 (ALDH1A1) [19]. ALDH1A1 expression correlated with drug resistance and tumor progression in breast cancer [20] and ovarian cancer [21] among others.

The present study aimed to examine the expression of PTPRK in ovarian cancer cell lines resistant to: CIS, PAC, DOX, TOP, VIN, and MTX and the impact of this molecule expression on total phosphotyrosine (pTYR) level and drug resistance. The second goal of this paper is to compare the relations between ALDH1A1 and PTPRK expression in development of drug resistance in ovarian cancer cell lines.

## 2. Results

### 2.1. Analyses of PTPRK Gene Expression in Drug-Resistant Ovarian Cancer Cell Lines

**Figure 1:**
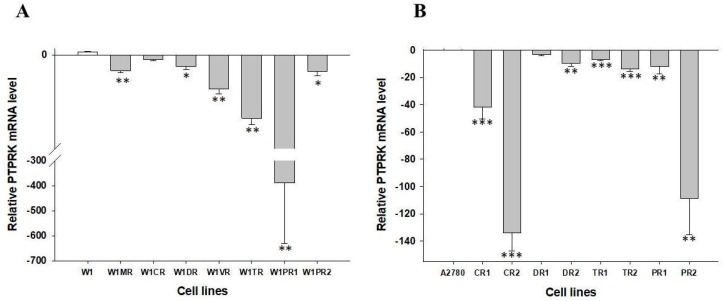
Expression analysis (Q-PCR) of the PTPRK gene in W1 and drug resistant cell lines (**A**) and A2780 and drug resistant cell lines (**B**). The figure presents the relative gene expression in the resistant cell lines (grey bars) with respect to that in the sensitive cell line (white bars), which was assigned a value of 1. The values were considered significant at * *p* < 0.05, ** *p* < 0.01 and *** *p* < 0.001.

The microarray data obtained before have suggested that changes in *PTPRK* gene expression can be involved in drug resistance in ovarian cancer [22]. To determine whether the development of drug resistance in W1 and A2780 drug-resistant sublines is associated with changes in *PTPRK* expression, expression of the *PTPRK* mRNA was assessed. We observed that the transcript level of *PTPRK* was significantly decreased in W1MR, W1DR, W1VR, W1TR, W1PR1 and W1PR2 cell lines (*p* < 0.05 or 0.01) (Figure 1A). However, substantial differences between investigated cell lines were observed. In most of the investigated cell lines, the transcript level was decreased from 5 to 20-fold. In contrast, in the W1PR1 cell line, we observed about 400-fold lower *PTPRK* mRNA level in a relation to the control. We also observed reduced expression level of *PTPRK* gene in A2780CR1, A2780CR2, A2780DR2, A2780TR1, A2780TR2, A2780PR1, and A2780PR2 cell lines (*p* < 0.01 or *p* < 0.001) (Figure 1B). According to the downregulation of *PTPRK* gene expression, we have divided drug resistant cell lines into three groups. Low downregulation (up to 15-fold: A2780DR2, A2780TR1, A2780TR2 and A2780PR1 cell lines), medium downregulation (up to 50-fold: A2780CR1 cell line) and high downregulation (over 100-fold: A2780CR2 and A2780PR2 cell lines). Expression according the drug specificity was as follows: *PTPRK* was downregulated in all four PAC-resistant cell lines, all three TOP-resistant cell lines, two from three CIS-resistant cell lines and two from three DOX-resistant cell lines. Additionally, downregulation was noted for one MTX and one VIN-resistant cell line.

### 2.2. Immunofluorescence Analysis of PTPRK and pTYR in Drug Sensitive and Resistant Cell Lines

**Figure 2:**
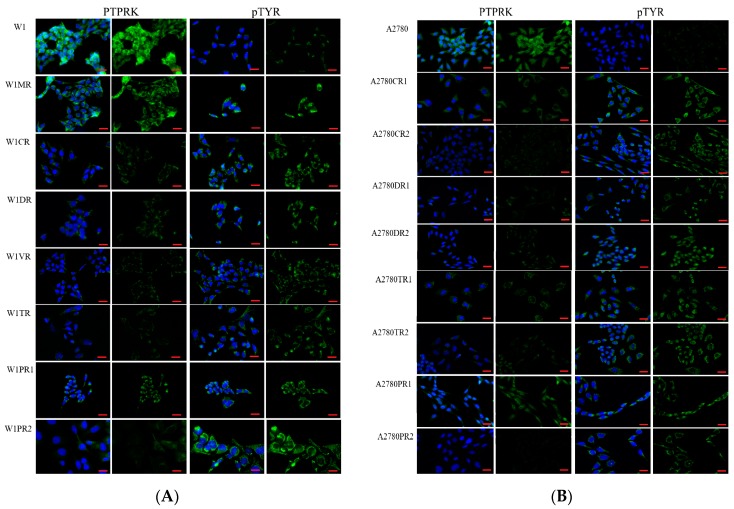
Immunofluorescence visualization of PTPRK and pTyr expression in the W1 and drug resistant cell lines (**A**) and in A2780 and drug resistant cell lines (**B**). PTPRK was detected using the anti-PTPRK antibody and an Alexa Fluor^®^488-conjugated secondary antibody (green). pTyr was detected using the anti-pTyr antibody and MFP488-conjugated secondary antibody (green). Cell nuclei were stained with DAPI (blue). Scale bar = 20 µm.

PTPRK is involved in pTYR removal from many proteins; therefore, we were interested whether changes in PTPRK expression correlate with pTYR level in investigated cell lines. Thus, we performed fluorescence analysis of PTPRK and pTYR at the cellular level in the investigated cell lines. We observed that in all W1 drug resistant cell lines the fluorescence signal of PTPRK was weaker when compared to control. In contrast, in all W1 drug resistant cell lines, an increased pTYR fluorescent signal was observed (Figure 2A). A similar pattern was noted for A2780 and respective drug resistant cell lines. All drug resistant A2780 cell lines showed a decreased fluorescent signal for PTPRK and an increased signal for pTYR (Figure 2B).

### 2.3. Western Blot Analysis of PTPRK and pTYR

**Figure 3:**
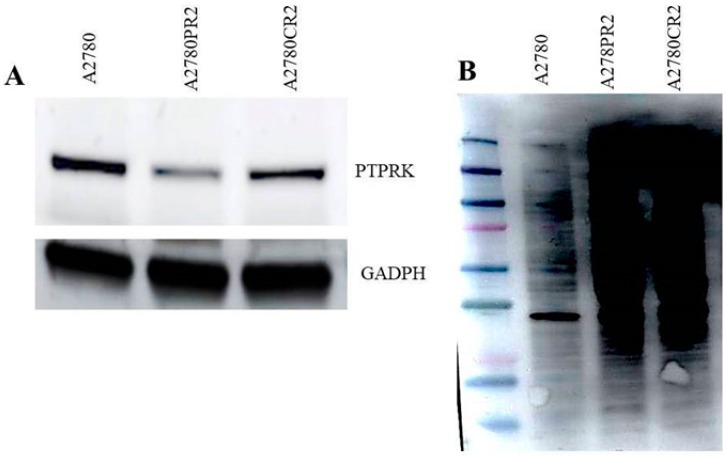
(**A**) PTPRK protein expression analysis in the A2780 and drug-resistant cell lines: A2780CR2 and A2780PR2. The cellular proteins were separated using 7% PAGE and transferred to a PVDF membrane, which was then immunoblotted with either primary antibody (Ab) or HRP-conjugated secondary Ab. A primary anti-GADPH Ab was used as a loading control for the cell lysates; (**B**) total pTYR protein expression analysis in the A2780 and drug-resistant cell lines A2780CR2 and A2780PR2. The cellular proteins and protein molecular weight marker were separated using 7% PAGE and transferred to a PVDF membrane, which was then immunoblotted with either primary Ab or HRP-conjugated secondary Ab.

The cellular expression of PTPRK observed in immunofluorescence analysis was confirmed by Western blot analysis for selected cell lines with high and low PTPRK expression level. In cell lysates from A2780CR2 and A2780PR2, a clear decrease in PTPRK bands intensity in comparison to parental drug sensitive cell line was noted (Figure 3A). This result corresponds with decreased transcript level and fluorescence signal in drug resistant cell lines. In contrast, drug resistant cell lines showed an increased signal for pTYR (Figure 3B), that remains in accordance with results of pTYR fluorescence analysis.

### 2.4. Early Response to Topotecan (TOP)-Treatment in Ovarian Cancer Cell Lines

**Figure 4:**
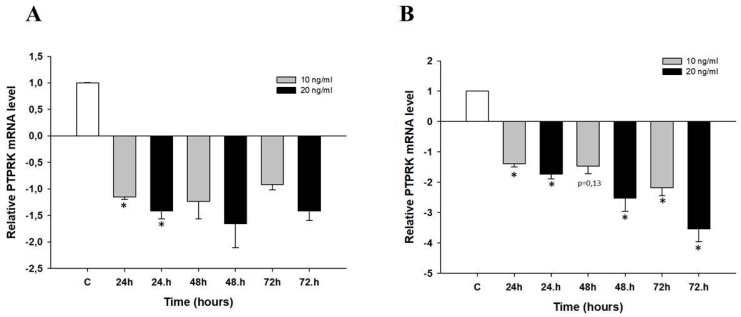
Expression analysis of *PTPRK* gene in W1 cell line (**A**) and A2780 cell line (**B**) after short time exposure to TOP treatment. The figure presents relative genes expression in TOP treated cells (grey and black bars) with respect to the untreated control (white bars) assigned as 1. The values were considered significant at * *p* < 0.05.

To check whether *PTPRK* gene is involved in early response to TOP treatment, investigated cell lines (W1 and A2780) were treated with low concentration of TOP (10 ng/mL and 20 ng/mL). After 24 h, 48 h and 72 h, gene expression was analyzed. In a W1 cell line, we observed downregulation of *PTPRK* gene expression after short time TOP treatment for all time and concentration points, although this downregulation was statistically significant only after 24 h (*p* < 0.05) (Figure 4A). In A2780 cell line statistically significant decreased expression of *PTPRK* was observed after 24 h, 48 h and 72 h incubation (*p* < 0.05), with the exception of 10 ng/mL after 48 h incubation where downregulation was close to significance (*p* = 0.13) (Figure 4B).

### 2.5. Co-Expression of PTPRK and ALDH1A1

**Figure 5:**
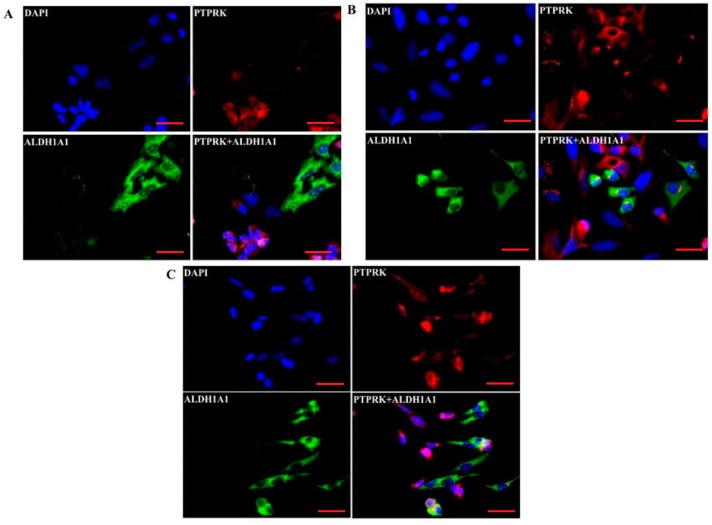
Immunofluorescence visualization of PTPRK and ALDH1A1 co-expression in the W1TR (**A**), W1PR2 (**B**) and A2780PR1 (**C**) cell lines. PTPRK was detected using the anti-PTPRK antibody and Alexa Fluor^®^594 secondary antibody (red). ALDH1A1 was detected using the anti-ALDH1A1 antibody and Alexa Fluor^®^488 secondary antibody (green). To visualize the cell nuclei, the cells were mounted with a DAPI-containing mounting medium (blue). Scale bar = 20 µm.

Immunofluorescence analysis revealed that PTPRK expression differs between cells within the same cell line. Previously, we have observed a similar pattern for ALDH1A1 expression analysis where subpopulation of ALDH1A1 positive cells was noted in W1TR [21], W1PR2 and A2780PR1 cell lines [23]. Hence, we were interested in whether there is any relation between PTPRK and ALDH1A1 expression. In W1TR cell lines, we observed three cell populations with medium, low and very low PTPRK expression. Cells expressing very low levels of PTPRK expressed very high levels of ALDH1A1, a marker of CSCs (Figure 5A). Similar cell populations with medium, low and very low PTPRK expression were observed in W1PR2 cell line. Cells with very low PTPRK expression expressed a very high level of ALDH1A1 (Figure 5B). In A2780PR1 cell lines, we observed two cell populations with medium and low PTPRK expression. Cells with lower levels of PTPRK presented higher ALDH1A1 levels (Figure 5C). Thus, in all three ALDH1A1 positive cell lines, expressions of PTPRK were noted as lowest in ALDH1A1 positive cells.

### 2.6. Immunohistochemistry

**Figure 6:**
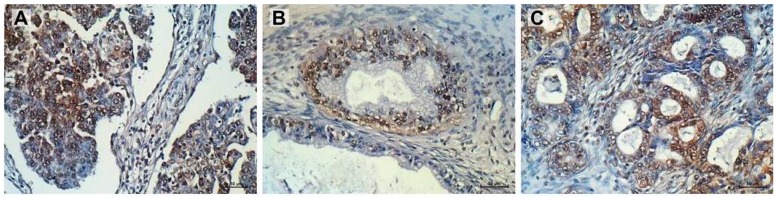
Immunohistochemical expression of PTPRK in serous adenocarcinoma (strong nuclear, moderate/weak cytoplasmic intensity score) (**A**); mucinous ovarian cancer (strong nuclear, moderate cytoplasmic intensity score) (**B**); and endometrioid adenocarcinoma (weak nuclear, strong cytoplasmic intensity score) (**C**). Sections were counterstained with hematoxylin. Scale bar = 50 μm.

Distinct types of ovarian cancer were immunohistochemically analysed in regard to verifying tissue expression of PTPRK. Few samples of serous, mucinous and endometrioid ovarian cancer were determined. A positive expression of PTPRK protein was observed regardless of the type of ovarian cancer. However, the distribution and strength of immunohistochemical signal differed among analysed tissues. In the serous adenocarcinoma, a majority of cancer cells presented a strong nuclear signal, whereas only in a part of them a moderate/weak cytoplasmic signal was observed (Figure 6A). In mucinous ovarian cancer specimens, a strong nuclear signal in almost all cancer cells was accompanied by a moderate cytoplasmic one (Figure 6B). On the contrary, in endometrioid adenocarcinoma, a weak nuclear signal with a strong cytoplasmic one was noticed (Figure 6C).

## 3. Discussion

The most important role that the treatment of ovarian cancer plays is cytoreductive surgery followed by chemotherapy [3]. However, like many types of the treatment, chemotherapy is not always efficient. The problem of drug resistance is common and still difficult to cope with [24].

Cancer cells develop different mechanisms of the drug resistance including: high expression of the drug efflux pumps [25], anti-apoptic molecules and DNA repair enzymes [24] or expression of different extracellular molecules leading to cell adhesion-mediated drug resistance (CAM-DR) [26].

Increased signal transduction resulting from imbalance between phosphorylation and dephoshorylation of tyrosine play a crucial role in altered gene expression leading to increased cell proliferation, malignant transformation and drug resistance [11,27,28]. The imbalance between those processes can result either from increased kinase activity [8] or from decreased phosphatases activity [9] and both of them are found to be affected in cancer cells. Although increased activity of kinases in cancer cells is abundantly described in literature, much less is known about the role of tyrosine phosphatases in cancer progression and drug resistance development. Decreased expression or activity of different phosphatases were already related to increased signal transduction and progression of different cancers [8,9].

Previously, we have developed and described a series of drug resistant ovarian cancer cell lines: W1 ovarian cancer cell line resistant to CIS, PAC, DOX, TOP, VIN and MTX [29] and A2780 cell line resistant to CIS, PAC, DOX, TOP (two cell line for each drug) [30]. All these cell lines were characterized in relation to drug transporters expression and cytotoxic drug cross-resistance [29,30], expression of extracellular matrix (ECM) molecules [23,31,32,33,34,35,36] as well as expression of “new genes” that can be related to drug resistance [37,38,39]. Some of the investigated cell lines also showed the presence of ALDH1A1 positive CSCs populations [21,23]. Among the “new genes” considered as related to drug resistance, the group of downregulated was noted and within those the *PTPRK* gene was found [22]. Hence, we focused in more detail on PTPRK expression in our drug resistance cell lines.

The result of our experiments demonstrated downregulation of the *PTPRK* gene followed by decreased protein expression in all investigated drug resistant cell lines. In contrast, the same cell lines showed an increased level of pTYR. Additionally, dose and time dependent decrease in *PTPRK* transcript level after incubation of drug sensitive cell lines with TOP was proved. The changed gene expression after short time exposure of drug sensitive cell lines to PAC, TOP or CIS [23,32,35,37,38,39] was observed by ours for other genes previously. These kinds of experiments are very rare because researchers mainly only compare gene expression between drug sensitive and resistant cell lines. It turns out that genes over- or under-expressed after the cancer cell had a short amount of contact time of with the drug remain at a high level later, when drug resistance develops. This further confirms the significance of the loss of PTPRK expression in drug resistance development.

Correlation between changes in PTPRK expression or activity and disease progression has been observed in several tumors. Sun et al. showed that a low level of *PTPRK* transcript is correlated with advanced breast cancer and poor prognosis. Additionally, knockdown of *PTPRK* in MDA-MB-231 and MCF-7 breast cancer cell lines resulted in increased cell proliferation, adhesion and invasion [15]. Similar results were also found in melanoma cells and tissue biopsies [40]. In primary central nervous system lymphomas (PCNSL), a tendency toward higher mortality was observed among patients with a loss of PTPRK expression [41]. Downregulation of PTPRK expression in nasal-type NK/T-cell lymphoma (NKTCL) correlated with advanced-stage disease and unfavorable prognosis. Restoration of PTPRK suppressed tumor cells proliferation and reduced migration and invasive ability of tumor cells. The level of PTPRK inversely correlated with phosphorylation and nuclear localization of transcription factor STAT3 and restoration of PTPRK decreased nuclear phospho-STAT3^TYR705^ level. [16].

*PTPRK* promoter methylation in acute lymphoblastic leukemia (ALL) also correlated with decreased overall survival and restoration of PTPRK expression resulted in reduced cells proliferation in Raji cell line. This probably resulted from downregulation of signaling proteins activity since reduction of phosphorylated extracellular signal-regulated kinases 1 and 2 (Erk 1/2), protein kinase B (Akt), STAT3 and STAT5 was observed in cells with PTPRK expression [42]. PTPRK was proved to be involved also in glioma progression since patients with deleted or inactivated *PTPRK* had poor overall survival when compared to those with normal PTPRK locus [43] and restoration of wild-type PTPRK (wt-PTPRK) in glioma cell lines resulted in reduced cells growth and migration. In glioma cells, PTPRK is involved in epidermal growth factor receptor (EGFR) and b-catenin dephosphorylation and loss of PTPRK activity results in increased EGFR and b-catenin phosphorylation [14].

In summary, decreased activity of PTPRK because of promoter inactivation or functional mutation was observed in different tumours and was mainly related to increased proliferation and migration of tumour cells since restoration of PTPRK had a tumour suppressive effect. In different cancers, increased phosphorylation of different signalling molecules was also observed [14,16,42]. In our study, we observed an increase in total pTYR level in our drug resistant cell lines. This further proves that downregulation of PTPRK leads to activity of many signal transduction pathways. However, decreased PTPRK expression on one hand and increased pTYR level on the other was not related to increased cells proliferation because our drug resistant cells showed a lower proliferation rate than drug sensitive cells W1 and A2780 (unpublished). Thus, changes in PTPRK expression in our drug resistant cell lines seem to be directly related to drug resistance development.

Correlation between PTPRK expression and response to cytotoxic chemotherapy was observed in several tumors. NKTCL patients with methylated PTPRK promoter had the worst response to therapy composed of dexamethasone, methotrexate, ifosfamide, L-asparaginase, and etoposide and shorter overall survival [16]. The acute lymphoblastic leukemia (ALL) cell lines Raji and Jurkat transfected with PTPRK vector were significantly more sensitive to AraC than cells transfected with empty vector [42]. In another study, U87-MG glioma cells transfected with wt-PTPRK were significantly more sensitive to temozolomide, erlotinib and gefitinib or combination of temozolomide with erlotinib or gefitinib than the same cells transfected with mutated, inactive form of PTPRK [14].

All of above-mentioned research suggests that loss of PTPRK activity results in higher resistance to different anticancer drugs. The results of our study confirm these findings as well. We have proved the loss of PTPRK expression in ovarian cancer cell lines resistant to six different cytotoxic drugs. Taken together, it could be assumed that decreased expression of PTPRK is not directly related to higher resistance to specific drugs but rather that is a general feature of drug resistant cells. That imbalance leads to changes in signal transduction, expression of genes involved in cell cycle and probably drug resistance development. The last one was already proved by ours, since many different proteins that are involved in drug resistance was described before. Among them, the most important role seems to play drug transporters from the ATP-binding cassette family (ABC family) and expression of P-gp related to PAC, DOX and VIN resistance and expression of BCRP related to TOP resistance in drug resistant cell lines was observed [29,30]. As it has been suggested that activity of drug transporters is regulated by phosphorylation event [44], especially direct role of phosphorylation in BCRP protein dimerization and activity has been described [45]. Thus, altered signal transduction may also lead to increased drug transporters activity along with increased expression of drug resistance genes in our drug resistant cell lines. Within the large family of kinases, there are enzymes (kinases of the Raf/MEK/ERK pathway) responsible for controlling the activity of membrane drug transporters. They phosphorylate the transcription factors that bind to the *MDR1* promoter region which increases its expression [27,46]. From the other hand, the loss of PTPRK stimulates the phosphorylation of ERK 1/2 [42]. In summary, the loss of PTPRK expression may be associated with increased drug resistance mediated by MDR1 responsible for resistance to PAC, VIN, DOX and TOP in investigated cell lines [29,30].

One another aspect of the role of PTPRK in the drug resistance development should be considered. As it was widely described, our drug resistant cell lines showed increased expression of different ECM molecules [23,31,32,33,34,35,36] that could be involved in cell adhesion mediated drug resistance (CAM-DR). Interaction of ECM molecules e.g., collagens with cell receptors, activates intracellular phosphoinositide-3-kinase–protein kinase B (PI3K/AKT) or mitogen-activated protein kinase (MAPK) pathways, which leads to the survival proteins’ activation [47]. Because AKT and ERK 1/2 are substrates for PTPRK [42], the loss of PTPRK expression may result in an activation of these pathways, resulting in increased genes expression and apoptosis resistance [26].

Previously, we observed the subpopulation of ALDH1A1 positive CSCs in ovarian cancer cell lines resistant to PAC and TOP [21,23]. The same cells showed also a high level of the drug transporters and some ECM molecules’ expression. In the present study, we were interested in whether there is any relation between expression of PTPRK and ALDH1A1 in these cell lines. Indeed, in all three cell lines, we observed a reversed correlation between ALDH1A1 and PTPRK expression, where ALDH1A1 positive population demonstrated the lowest level of PTPRK expression. Thus, we confirm that loss of PRPRK expression correlates with development of drug resistance and may influence the decline in CSCs which expresses many of drug resistant proteins [19]. To our knowledge this is the first observation where relation between PTPRK and ALDH1A1 expression has been demonstrated and emphasize the role of PTPRK loss in tumor development.

In this light, two options could be considered: (1) Cancer cells present different levels of PTPRK expression. Cells with low PTPRK expression are more resistant to chemotherapy because of more active signal transduction survival pathways and these cells survive after contact with cytotoxic agent. (2) In response to cytotoxic drug treatment, cells reduce the PTPRK expression, which leads to activation of survival pathways resulting in drug resistance gene expression and resistance to chemotherapy. At present, it remains difficult to say which hypothesis is true. To definitely confirm the significance of losing PTPRK expression in drug resistance development, more detailed experiments are required. In particular, identification of signaling proteins activated as a result of loss of PTPRK expression seems to be crucial.

The final step of our research was to examine PTPRK expression in ovarian cancer tissue. Several cases of different ovarian cancer subtypes were tested and all of them showed positive signal for PTPRK expression. However, the distribution and strength of immunohistochemical staining in our study were different and dependent on the type of ovarian cancer. The strongest nuclear signal was noted for cancer cells of serous adenocarcinoma and mucinous ovarian cancer, whereas endometrioid adenocarcinoma showed the weak nuclear accompanied by a strong cytoplasmic signal. To our knowledge, this is the first observation of PRPRK expression in ovarian cancer. However, expression of PTPRK was observed in some other cancers. In breast cancer, PTPRK staining was stronger in tumor tissue compared to control, and patients with higher *PTPRK* mRNA levels presented higher overall and progression free survival [15]. In NKTCL tumors, an inverse correlation between PTPRK and pSTAT3 was observed [16]. Expression of PTPRK in ovarian cancer suggests its involvement in cancer progression; however, to further explain the significance of PTPRK in tumor development, a big cohort of patients should be analyzed.

## 4. Materials and Methods

### 4.1. Reagents and Antibodies

Cytotoxic drugs: MTX, CIS, DOX, VIN, TOP and PTX as well as RPMI-1640 and MEM medium, FBS, antibiotic-antimycotic solution, and L-glutamine were acquired from Sigma (St. Louis, MO, USA). Mouse monoclonal anti-PTPRK Ab (sc-374315), anti-p-Tyr (sc-508), donkey anti-goat horseradish peroxidase- (HRP) conjugated Ab and mounting medium with DAPI were obtained from Santa Cruz Biotechnology (Santa Cruz, CA, USA). Rabbit monoclonal anti-ALDH1A1 Ab (ab52492) was purchased from Abcam, Cambridge, UK. The fluorescent secondary antibodies: MFP488 Goat Anti-Mouse IgG were obtained from MoBiTec (Goettingen, Germany) and Alexa Fluor^®^488 Donkey Anti-Rabbit and Alexa Fluor^®^594 Donkey Anti-Mouse IgG respectively were obtained from Jackson ImmunoResearch Laboratories (Cambridgeshire, UK).

### 4.2. Cell Lines and Cell Culture

The established ovarian cancer cell lines A2780 and the primary ovarian cancer cell line W1 were used in this study. A2780 were purchased from ATCC (American Type Culture Collection, Manassas, VA, USA). The resistant A2780 cells were generated by exposing cells to the cytotoxic drugs: CIS, DOX, TOP, PAC at incrementally increasing concentrations. In this way, resistant A2780 sublines were obtained: A2780CR1 and A2780CR2 (A2780 cisplatin resistant), A2780DR1 and A2780DR2 (A2780 doxorubicin resistant), A2780TR1 and A2780TR2 (A2780 topotecan resistant), A2780PR1 and A2780PR2 (A2780 paclitaxel resistant). Ovarian cancer tissue from an untreated patient was used to establish a W1 human ovarian cancer cell line. Exposing W1 cells to the cytotoxic drugs: MTX, CIS, DOX, VIN, TOP, PAC at incrementally increasing concentrations allowed for generating resistant W1 cells: W1MR (W1 methotrexate resistant), W1CR (W1 cisplatin resistant), W1DR (W1 doxorubicin resistant), W1VR (W1 vincristine resistant), W1TR (W1 topotecan resistant), W1PR1 and W1PR2 (W1 paclitaxel resistant). The final concentrations used for selecting the resistant cells were: 28 ng/mL of MTX, 1000 ng/mL of CIS, 100 ng/mL of DOX, 10 ng/mL of VIN, 24 ng/mL of TOP and 1100 ng/mL of PAC.

The increase in resistance according to parental drug sensitive cell line A2780 were as follows: 4.1-fold for A2780CR1 vs. A2780; 3.3-fold for A2780CR2 vs. A2780; 58.0-fold for A2780DR1 vs. A2780; 73.0-fold for A2780DR2 vs. A2780; 59.6-fold for A2780TR1 vs. A2780 and 48.5-fold for A2780TR2 vs. A2780; 146-fold for A2780PR1 vs. A2780 and 1202-fold for A2780PR2 vs. A2780 as described previously [29]. The increase in resistance according to parental drug sensitive cell line W1 were as follows: 138-fold for W1MR vs. W1; 7.9-fold for W1CR vs. W1; 10.3-fold for W1DR vs. W1; 24.5-fold for W1VR vs. W1; 20-fold for W1TR vs. W1; 641-fold for W1PR1 vs. W1 and 967-fold for W1PR2 vs. W1 as described previously [30,48].

All of the cell lines were harvested in medium enriched in FBS (10% *v*/*v*), L-glutamine (200 mM), antibiotics: penicillin and streptomycin (100 units/mL) and amphotericin B (25 μg/mL)]. RPMI-1640 medium was used for W1 cells and MEM medium was used for A2780 cells. Cells were grown at 37 °C in a 5% CO_2_ atmosphere.

### 4.3. Examination of Gene Expression Using Q-PCR

The changes in *PTPRK* gene expression in the W1, A2780 and drug resistant cell lines were examined. The Gene Matrix Universal RNA purification Kit (EURx Ltd. Gdansk, Poland) was used to isolate RNA. This procedure was carried out in accordance with the manufacturer’s protocol. Reverse transcription was performed using the M-MLV reverse transcriptase (Invitrogen by Thermo Fisher Scientific, Waltham, MA, USA) according to the manufacturer’s protocol and using a thermal cycler (Veriti 96 well Thermal Cycler, Applied Biosystems, Foster City, CA, USA). Two µg of RNA were used to synthesize cDNA. Maxima SYBR Green/ROX qPCR Master Mix (Thermo Fisher Scientific, Waltham, MA, USA) and sequence-specific primers from Oligo.pl (Warsaw, Poland) (Table 1) were used for Real-time PCR. 12.5 μL of Maxima SYBR Green/ROX qPCR Master Mix, 1 μL of each primer, 9.5 μL of water, and 1 μL of cDNA solution were mixed together. As the negative control, the sample without cDNA was used. The analysis was conducted on the Applied Biosystems PCR System (7900HT Fast Real-Time PCR System, Foster City, CA, USA) following the PCR protocol: initial denaturation 95 °C for 15 min, 45 cycles of denaturation at 95 °C for 15 s, annealing at 60 °C for 30 s, and extension at 72 °C for 30 s. Internal reference controls were used including: β-actin, beta-2-microglobulin (β2M), hypoxanthine-guanine phosphoribosyltransferase 1 (HRPT1) and glyceraldehyde-3-phosphate dehydrogenase (GADPH). Product melting temperature was analyzed and to check the quality and quantity of the amplification products, gel electrophoresis in 3% agarose with ethidium bromide was performed.

To analyze tested genes, the relative quantification (RQ) method was used with reference to drug sensitive cell lines (W1, A2780) as the calibrators (RQ of the calibrator = 1) The analysis was conducted using the following standard formula: RQ = 2 − ΔΔCt (where ΔΔCt = ΔCt of the sample (drug-resistant line) − ΔCt of the calibrator (drug sensitive line)). The graphs were plotted using Sigma Plot (Systat Software, Inc., San Jose, CA, USA).

### 4.4. Protein Isolation from Cell Culture

RIPA buffer containing protease inhibitor cocktail (Roche Diagnostics GmbH, Mannheim, Germany) was used to lyse the cells (1 × 10^6^ cells/25 μL lysis buffer). The process of the lysis was carried out for 60 min on ice at 4 °C The Bradford protein assay system (Bio-Rad Laboratories, Hemel Hempstead, UK) was used to determine the concentration of the protein.

### 4.5. SDS-PAGE and Western Blot Analysis of PTPRK and p-Tyr

For Western Blot analysis, thirty micrograms protein from each sample were resuspended in 4x loading buffer (Bio-Rad Laboratories, Hemel Hempstead, UK) and incubated for 20 min at RT. Afterwards, samples were separated on a 4–20% mini- PROTEAN^®^ TGX™ precast gel (Bio-Rad Laboratories, Hercules, CA, USA), using the SDS-PAGE technique and transferred to a nitrocellulose membrane. Membrane with proteins was blocked with 5% milk in Tris-Buffered Saline (TBS)/Tween (0.1 M Tris-HCl, 0.15 M NaCl, 0.1% Tween 20). Immunodetection of an interesting protein was undertaken using rabbit anti-PTPRK Ab at 1:1000 dilution and the appropriate HRP-conjugated secondary Ab. Separated bands were visualized by chemiluminescence detection using chemical luminescence (ECL) kit (Femto Super Signal Reagent, Thermo Fisher Scientific Inc., Waltham, MA, USA) and Hyperfilm ECL from Amersham (Piscataway, NJ, USA). To check correct protein loading in the lines, the stripping of membrane was performed. Reblotting was done with rabbit anti-GAPDH Ab (Santa Cruz Biotechnology, CA, USA) at 1:1000 dilution and goat anti-rabbit HRP-conjugated Ab.

The samples for Western Blotting of phospho-proteins were prepared and resuspended according to the protocol above. The proteins were transferred to a PVDF membrane, blocked with 5% w/v bovine serum albumin (BSA) in Tris-Buffered Saline with 0.5% Tween 20 (TBST) and immunodetected using mouse anti-pTyr Ab at 1:200 dilution and the appropriate HRP-conjugated secondary Ab. The visualisation of the results was carried out as above.

### 4.6. Immunofluorescence Analysis

Immunofluorescence analysis was performed on cells growing on microscopic glass slides according to previously established protocol [44]. For detecting the expression and localisation of PTPRKand/orp-Tyr, cells were fixed and permeabilized with ice-cold acetone/methanol (1:1) for 10 min, rinsed with PBS and blocked in 3% BSA for 30 min at room temperature. After that, cells were incubated for 2 h at room temperature with primary antibodies, anti-PTPRK (mouse monoclonal anti-PTPRK antibody, 1:200, Santa Cruz, CA, USA) and anti-p-Tyr (mouse monoclonal anti-pTyr antibody, 1:200 Santa Cruz, CA, USA). Next, cells were washed with PBS and incubated with the secondary antibody solution for 1 h at room temperature (for PTPRK-Alexa Fluor^®^488 Donkey Anti-Rabbit IgG, Jackson ImmunoResearch Laboratories, Cambridgeshire, UK and for p-Tyr-MFP488 Goat Anti-Mouse IgG, Goettingen, Germany). Afterwards, slides were washed with PBS and sealed with DAPI-containing mounting medium. The analysis of immunofluorescence expression was prepared with the use of fluorescence microscope (Zeiss Axio-Imager.Z1, Oberkochen, Germany).

### 4.7. Double Immunofluorescence Analysis

The initial conditions of double fluorescence staining (fixation, blocking and washing steps) were as described for immunofluorescence procedure. After the blocking step, the incubation with primary antibodies proceeded with the mixture of anti-PTPRK and ALDH1A1 antibodies simultaneously (2h, RT). The next step, followed by washing with PBS, was incubation in the mixture of two respective green dye-labelled (Alexa Fluor^®^488, donkey anti-rabbit IgG, 1:400 Jackson ImmunoResearch Laboratories, Cambridgeshire, UKand red dye-labelled (Alexa Fluor^®^594, donkey anti-mouse IgG, 1:400, Jackson ImmunoResearch Laboratories, Cambridgeshire, UK) secondary antibodies, for 1 h at room temperature. Afterwards, the cells proceeded the same way as for immunofluorescence protocol.

### 4.8. Incubation of Cells with TOP

Drug sensitive ovarian cancer cell lines (W1 and A2780) were seeded into 6-well plates at 0.5 × 10^6^ in 1 mL of medium per well in 6-well plates. The next cells were incubated with TOP in two concentrations: 10 ng/mL and 20 ng/mL for 24, 48 and 72 h. After treatment with TOP, cells were harvested and RNA was isolated.

### 4.9. Immunohistochemistry

Immunohistochemical staining was performed for formalin-fixed, paraffin embedded human ovarian carcinoma patient samples according to standard procedure. Briefly, sections were dewaxed with xylene, gradually hydrated, blocked in normal goat serum for 30 min and incubated with anti-PTPRK primary antibody (1:200) overnight at 4 °C followed by incubation with EnVision Detection System (Dako REALTM EnVisionTM Detection System peroxidase/DAB+, Rabbit/Mouse, Dako, Glostrup, Denmark) for 30 min at room temperature. The sections were then washed, counterstained with haematoxylin, dehydrated and mounted. Analysis of immunohistochemical staining was taken under the optical Olympus BH-2 microscope (Olympus America Inc., Center Valley, PA, USA) coupled to a digital camera.

The expression of PTPRK was calculated for 10 microscope fields at magnification of 400×, taking into account the mean proportion of immunopositive cancer cells among all cancer cells. For each specimen, the expression score was evaluated using the semi-quantitative scale with less than 10% of cancer cells weakly positive scored as 0 (negative); 11% to 50% positive cancer cells scored as 1 (weak); 51% to 75% positive cancer cells scored as 2 (moderate); and up to 75% positive cancer cells scored as 3 (strong).

### 4.10. Statistical Analysis

Data management and analysis were performed using Microsoft Excel software (2016, Available online: https://microsoft-excel-2016.softonic.pl). Significance levels were set using the Student *t*-test.

## 5. Conclusions

In summary, according to our knowledge, the presented study is the first report about PTPRK expression in drug resistant cell lines and in ovarian cancer tissue. The research has found decreased level of PTPRK expression in ovarian cancer cell lines resistant to CIS, PAC, DOX, TOP, VIN and MTX. Downregulation seem to be not related to specific drug resistance but is rather a general feature of drug resistant cells. Additionally, the lower PTPRK expression was observed in ALDH1A1 positive cell population, suggesting the role of PTPRK downregulation in primary as well as acquired resistance to cytotoxic drugs.

The results of the present study may improve our understanding of PTPRK in mechanisms leading to drug resistance in ovarian cancer. However, the significance of PTPRK expression downregulation in drug resistant cell lines and in ovarian cancer development requires further investigation and should be confirmed in ovarian cancer cell lines developed from different histological types of cancer, and for a larger group of patients.

## Figures and Tables

**Table 1:** Oligonucleotide sequences used for Real-Time Quantitative Polymerase Chain Reaction (RQ-PCR) analysis.

Transcript	Sequence (5′–3′ Direction)	Ensembl Transcript (ENST) Number (Available Online: http://www.ensembl.org)	Product Size (bp)
PTPRK	CCCAGGACCTCCACTAATCA ATTCCCAGTCCACAGCAATC	00000368226	110 bp
GADPH	GAAGGTGAAGGTCGGAGTCA GACAAGCTTCCCGTTCTCAG	00000229239	199 bp
β-actin	TCTGGCACCACACCTTCTAC GATAGCACAGCCTGGATAGC	00000331789	169 bp
HRPT1	CTGAGGATTTGGAAAGGGTG AATCCAGCAGGTCAGCAAAG	00000298556	156 bp
β2M	CGCTACTCTCTCTTTCTGGC ATGTCGGATGGATGAAACCC	00000558401	133 bp

## Abbreviations

PTPRK	Protein Tyrosine Phosphatase Receptor Type K
CSCs	Cancer Stem Cells
CR	Cisplatin Resistance
DR	Doxorubicin Resistance
MR	Methotrexate Resistance
PR	Paclitaxel Resistance
TR	Topotecan Resistance
VR	Vincristine Resistance
ALDH1A1	Aldehyde Dehydrogenase 1 Family Member A1
